# Deep (Meta)genomics and (Meta)transcriptome Analyses of Fungal and Bacteria Consortia From Aircraft Tanks and Kerosene Identify Key Genes in Fuel and Tank Corrosion

**DOI:** 10.3389/fmicb.2021.722259

**Published:** 2021-10-01

**Authors:** Ines Krohn, Lutgardis Bergmann, Minyue Qi, Daniela Indenbirken, Yuchen Han, Pablo Perez-Garcia, Elena Katzowitsch, Birgit Hägele, Tim Lübcke, Christian Siry, Ralf Riemann, Malik Alawi, Wolfgang R. Streit

**Affiliations:** ^1^Department of Microbiology and Biotechnology, Institute of Plant Science and Microbiology, University of Hamburg, Hamburg, Germany; ^2^Bioinformatics Core, University Medical Center Hamburg-Eppendorf, Hamburg, Germany; ^3^Virus Genomics, Heinrich-Pette-Institute, Leibniz Institute for Experimental Virology, Hamburg, Germany; ^4^Institute of General Microbiology, Molecular Microbiology, Kiel University, Kiel, Germany; ^5^Faculty of Medicine, Core Unit Systems Medicine, University of Würzburg, Würzburg, Germany; ^6^T/TQ-MN, Lufthansa Technik AG HAM, Hamburg, Germany

**Keywords:** fuel contamination, biofilm formation, biocorrosion, biofouling, bacteria and fungi, omics analysis, kerosene and aircraft

## Abstract

Microbial contamination of fuels, associated with a wide variety of bacteria and fungi, leads to decreased product quality and can compromise equipment performance by biofouling or microbiologically influenced corrosion. Detection and quantification of microorganisms are critical in monitoring fuel systems for an early detection of microbial contaminations. To address these challenges, we have analyzed six metagenomes, one transcriptome, and more than 1,200 fluid and swab samples taken from fuel tanks or kerosene. Our deep metagenome sequencing and binning approaches in combination with RNA-seq data and qPCR methods implied a metabolic symbiosis between fungi and bacteria. The most abundant bacteria were affiliated with α-, β-, and γ-Proteobacteria and the filamentous fungi *Amorphotheca*. We identified a high number of genes, which are related to kerosene degradation and biofilm formation. Surprisingly, a large number of genes coded enzymes involved in polymer degradation and potential bio-corrosion processes. Thereby, the transcriptionally most active microorganisms were affiliated with the genera *Methylobacteria, Pseudomonas, Kocuria, Amorpotheka, Aspergillus, Fusarium, and Penicillium*. Many not yet cultured bacteria and fungi appeared to contribute to the biofilm transcriptional activities. The largest numbers of transcripts were observed for dehydrogenase, oxygenase, and exopolysaccharide production, attachment and pili/flagella-associated proteins, efflux pumps, and secretion systems as well as lipase and esterase activity.

## Introduction

Microbial contamination of fuels is a phenomenon widely reported in the literature. Microbiological analyses showed that fuel systems like aviation fuel and storage tanks are often contaminated by a variety of microorganisms with a rather low diversity (Gaylarde et al., 1999; Rauch et al., 2006; Yemashova et al., 2007). In fact, detailed knowledge of the physiology and metabolism of complex bacterial–fungal communities in fuel-associated environments is limited. Previous studies implied that the bacteria–fungi microbial communities can form tightly attached biofilms on various surfaces. These studies identified mainly microorganisms associated with the bacterial genera *Methylobacterium, Bacillus, Pseudomonas, Serratia, Sphingomonas, Variovorax, Flavobacterium, Arthrobacter, and Alcaligenes*, as well as a limited number of fungal isolates linked to Amorphotheca (also known as Cladosporium and Hormoconis), Aspergillus, and Fusarium; no archaea were reported (Beech and Sunner, 2004; Beale et al., 2014; Khan et al., 2015).

Crude oil and derived products consist of hydrocarbons of various molecular weights and other organic compounds. Fuel products are refined alkanes, alkenes, and cycloalkanes of different chain lengths as well as aromatic compounds, especially benzene, toluene, and xylene. In general, fuel products are subject to microbial degradation (i.e., biofouling), which affects mostly kerosene (aviation fuel) and diesel fuels. Thereby, microbial alkane degradation is important for the bioremediation of petroleum-contaminated environments as well as for microbial enhanced oil recovery (Gaylarde et al., 1999; Brown et al., 2010).

Bacterial and fungal biocatalytic oxidation and degradation reactions of alkanes, alkenes, and aromatics are mediated by different enzymes such as dehydrogenases, laccases, and peroxidases (Clemente et al., 2001; Saratale et al., 2007; Khan et al., 2015). By utilizing fuels as a C-source and by metabolite excretion, microorganisms cause fuel alteration and deterioration. Other effects of microbial contamination are biofouling/biodegradation and corrosion of fuel system components such as pipelines, filters, storage tanks, and effects of the protective layer of paint on the tank surfaces (Rauch et al., 2006; Passman, 2013; Li et al., 2017; Martin-Sanchez et al., 2018). Under aerobic conditions, the utilization of the hydrocarbons of diesel fuel and aviation kerosene by microorganisms results in the production of organic acids, which are corrosive to metals (Hendey, 1964; Parbery, 1969; Morton and Surman, 1994). Under anaerobic conditions, sulfate-reducing bacteria and the expression of the dissimilatory sulfate reductase (DsrA, DsrB) are a major cause of corrosion in low-oxygen or oxygen-free environments (King and Miller, 1971; Gaylarde and Beech, 1989; Morton and Surman, 1994).

Initial studies suggests that polymeric coatings, which are designed to prevent contact of the underlying materials with corrosive media and microorganisms, were found to be susceptible to microbial degradation after formation of microbial biofilms on the surfaces of the coating materials, including epoxy and polyamide primers and aliphatic polyurethanes, exposing the underlying metals to biofilm-enhanced bio-corrosion (Thorp et al., 1997; Gu et al., 1998; Ii et al., 1998; Stern and Howard, 2000). Notably, in general, the overall degradation pathways and microbial interactions have yet to be fully researched. Within this framework, we addressed the following questions in the current study: Can we identify specific bacterial communities associated with fungi in different aircraft habitats and fuel-containing biofilms? What are the most active genes in these communities, and do they give any hints on biofouling of kerosene and bio-corrosion processes? To answer these questions, we first systematically characterized the microbiomes of six biofilms grown directly in aircraft tanks. We used these data to map transcriptomes of one fresh kerosene biofilm. In addition, we have analyzed more than 1,200 fluid and swab samples to evaluate the diversity of bacteria and fungi. This comprehensive data set gives a detailed insight into the community structure and the most strongly expressed genes. Further, it offers first clues on biofouling and the initial corrosion of the tank and protective paint.

## Materials and Methods

### Sampling

#### Metagenome/Metatranscriptome Samples

Fuel biofilm and liquid samples were taken from different suppliers and were provided via the Lufthansa Basis in Hamburg, Germany. Samples were collected from right-hand, left-hand, main, center, and reserve tanks mainly (Supplementary Table 1). Samples 1–3 for the metagenomic studies represent biofilm samples of planes mostly based in the equatorial climate zone. Samples 4–6 are derived from planes based in the central European climate zone. Samples for quantitative PCRs were obtained from fuel samples and only few biofilm samples or water samples (Supplementary Table 1). For total RNA isolation, the samples were taken by the use of the “DNA/RNA Shield Collection Tube w/Swab” (R1107-E, Zymo Research, Freiburg, Germany) and stored at –80°C for further analysis.

#### Microbial Community Analyses

In addition, we analyzed > 1,200 kerosene, water/kerosene, and biofilm samples (Supplementary Table 1). The samples were all collected from airplanes made by global manufacturers and were obtained from the Lufthansa Basis in Hamburg, Germany. Liquid samples were first sonicated by the use of a Handheld Ultrasonic Homogenizer UP200Ht (200 W, 26 kHz) (Hielscher Ultrasonics GmbH, Teltow, Germany) following the manufacturer’s description. Sonification was done for a maximum of 30 s using an amplitude of 60% and a cycle condition of 0.5. One hundred milliliters was filtrated using a 0.22-μm cellulose-acetate filter (Typ 11107, Sartorius Stedim Biotech, Göttingen, Germany).

### Scanning Electron Microscopy

Scanning electron microscopy (SEM) was performed as previously published (Krohn-Molt et al., 2013, 2017). Therefore, samples were fixed in paraformaldehyde (1%) (30525-89-4, Merck, Taufkirchen Deutschland) and glutaraldehyde (0.25%), (G4004, Merck, Taufkirchen Deutschland), dehydrated by ascending alcohol series and dried at the critical point with Balzers CPD 030 Critical Point Dryer (BAL-TEC, Schalksmühle, Germany). After coating samples with gold/carbon using a sputter coater SCD 050 (BAL-TEC, Schalksmühle, Germany), scanning electron micrographs were taken with a LEO 1525 (Zeiss, Oberkochen, Germany).

### Total DNA Extraction From Biofilm and Liquid Samples

To obtain highly purified DNA, different isolation kits were tested. Finally, we settled that, for the metagenome analyses, high-quality DNA was extracted from the filters and cotton swabs by using the “NucleoBond High Molecular Weight Genomic DNA-Kit” with minor modifications (740160.20, MACHEREY-NAGEL, Düren, Germany). For the analyses via qPCR, the DNA was extracted via the “Quick DNA Fungal/Bacterial Microprep-Kit” (D6007, Zymo Research, Freiburg, Germany) with the addition of 5 mg lysozyme and 0.5 mg proteinase K (89833 and EO0491, Thermo Scientific^TM^, Darmstadt, Germany). Extracted DNA was stored at 4°C overnight. The concentration and purity of DNA were analyzed using a NanoPhotometer^®^ NP80 (IMPLEN, München, Germany).

### Metagenome Sequencing, *de novo* Assembly, and Binning

DNA Libraries were constructed applying the NEBNext^®^ Ultra^TM^ DNA Library Prep Kit for Illumina (E7370L, New England Biolabs, Frankfurt am Main, Germany), according to the manufacturer’s protocol, which is based on ligation of Illumina adapters. Depending on sample concentration, 3–60 ng DNA was used. The initial fragmentation of DNA was performed on the Bioruptor^®^ NGS [Diagenode, Seraing (Ougrée), Belgium] with 30 s on/30 s off for 16 cycles. Sequencing of metagenomic DNA libraries was performed on the NextSeq 500 platform (Illumina, San Diego, CA, United States) as paired-end run (2 × 150 cycles) with approximately 33–168 mio reads per sample.

The quality of raw sequencing data was assessed using FastQC (v0.11.8, Andrews, 2010). The sequences of sequencing adapters and low-quality bases (Q-score < 20) were removed using Trimmomatic (v0.39, Bolger et al., 2014). Kraken2 (v2.0.7-beta, Wood and Salzberg, 2014) was used in combination with the NCBI nt database (obtained on October 27, 2019) for the k-mer-based taxonomic classification. Relative abundances were then estimated with Bracken (v2.2, Lu and Salzberg, 2020). Complementarily, an assembly *based* analysis was carried out. Trimmed sequence reads were assembled with metaSPAdes (v3.14.0, Nurk et al., 2017). Contigs shorter than 1,000 bps were not used in subsequent analysis steps. Sequence reads were aligned to contigs using BWA mem (v0.7.17, Li, 2013) for abundance estimation and subsequent binning with metaBAT2 (v2.12.1, Kang et al., 2019). The binned sequences were aligned to the nt database using blastn of the BLAST + suite (v2.7.1) in megablast mode (Camacho et al., 2009). The alignment results were used to manually curate the binning results.

For the sequences’ functional characterization and additional 16S rRNA phylogenetic profiling, the Integrated Microbial Genomes (IMG) pipeline and homology searches was used (Chen et al., 2019; Mukherjee et al., 2019).

### RNA Extraction and Sequencing

Triplicates of the biofilm sample were used for total RNA extraction. Therefore, we used a hot phenol method with minor modifications previously published (Krohn-Molt et al., 2017). The concentration and quality of the total RNA were measured by a NanoDrop ND 2000 instrument (PEQLAB Biotechnologie GmbH, Erlangen, Germany) and verified on a 1.2% formaldehyde-agarose gel. For the preparation of DNA libraries suitable for sequencing, ∼300 ng of total RNA was subjected to rRNA depletion using Illumina’s Ribo-Zero Gold Epidemiology kit (MRZE724, Illumina, San Diego, CA, United States). After rRNA depletion, RNA samples were fragmented with Mg2 + at 94°C for 3 min using the NEBNext Magnesium RNA Fragmentation Module (E6150S, New England Biolabs, Frankfurt am Main, Germany) followed by RNA purification with the Zymo RNA Clean & Concentrator kit (R1019, Zymo Research, Freiburg, Germany). Fragmented RNA was dephosphorylated at the 3′ end, phosphorylated at the 5′ end, and decapped using 10 U T4-PNK ± 40 nmol ATP and 5 U RppH, respectively (M0356S, New England Biolabs, Frankfurt am Main, Germany). After each enzymatic treatment RNA was purified with the Zymo RNA Clean & Concentrator kit. The small RNA fragments were ligated for cDNA synthesis to the 3′ SR and 5′ SR adapter using the NEBNext Multiplex Small RNA Library Prep kit for Illumina (E7560S, New England Biolabs, Frankfurt am Main, Germany). PCR amplification to add Illumina adaptors and indices to the cDNA was performed for 16 cycles. Barcoded DNA Libraries were purified using magnetic MagSi-NGSPREP Plus beads (MDKT00010500, AMS Biotechnology, Abingdon, United Kingdom). Libraries were quantified with the Qubit 3.0 Fluometer (Thermo Scientific^TM^, Darmstadt, Germany), and library quality and size distribution were checked on a 2100 Bioanalyzer with the high-sensitivity DNA kit (Agilent, Waldbronn, Germany). Sequencing was performed with 5% PhiX Control Library as spike-in on the NextSeq 500 platform (Illumina, San Diego, United States) in 2 × 150 nt paired-end mode with the High Output Kit v2.5 (300 cycles). Demultiplexed FASTQ files were generated with bcl2fastq2.

### Processing and Analysis of RNA-seq Reads

Quality assessment was performed with FastQC as described above. Quality control was performed with fastp (v0.20.0, Chen et al., 2018); paired-end reads were corrected and merged in this step (with parameters –merge –correction). The merged reads were then processed with CoMW for functional annotation (v1.0.0, Anwar et al., 2019). During this step, reads were assembled and aligned to integrated databases M5nr and eggNOG (v3.0) (with parameter -d 1) (Powell et al., 2012; Wilke et al., 2012). Contigs assembled by CoMW were aligned to the NCBI nt database used as previously described (Gallardo-Becerra et al., 2020). Mapping of RNA reads to the reference genomes of *Methylobacterium brachiatum* (GCF_003697185.1), *Amorphotheca resinae* (GCA_003019875.1), and the *de novo*-assembled transcriptomes was performed with Bowtie2 v2.3.5.1 (Langmead et al., 2019) and TopHat2 v2.1.1 (Kim et al., 2013). Htseq-count v0.13.5 (Anders et al., 2015) was used to calculate the number of reads mapping to each feature in the genomes. Subsequently, RPKM values were calculated for each feature and circular representations of both genomes in Figure 6 were constructed with DNAPlotter (Carver et al., 2009), included in Artemis v17.0.1 (Carver et al., 2012). Log2-normalized values were calculated against the ‘‘housekeeping’’ sigma factor 70-coding rpoD (EBB05_11765) for *M. brachiatum* and transcription factor TFIIB (M430DRAFT_41748) for *A. resinae*. In order to overcome the lack of annotation for the genome of *A. resinae*, all encoded protein sequences were searched locally against NCBI’s non-redundant database^1^ with DIAMOND blastp v0.9.30.131 (Buchfink et al., 2015). All data are found in Supplementary Table 6.

### qPCR Procedures and Quantification

The number of 16S rRNA and ITS-region gene copies was determined for all samples by real-time PCR. The analysis was performed by the QuantStudio 3 detection system by the use of SYBR^®^ Select Master Mix (4472919, Thermo Scientific^TM^, Darmstadt, Germany) and the primer bact_338f/518r (5′-ACT CCT ACG GGA GGC AGC AG-3′, 5′-ATT ACC GCG GCT GCT GG-3′; Ovreås et al., 1997; Ishii and Fukui, 2001) and fungi_NL1f/LS2r (5′-ATA TCA ATA AGC GGA GGA AAAG-3′, 5′-ATT CCC AAA CAA CTC GAC TC-3′; O’Donnell, 1993; Cocolin et al., 2000). Each reaction contained 0.15 ng/μl of template DNA, 1.25 μM of each primer (Eurofins, Hamburg, Germany), and one-time SYBR^®^ Select Master Mix. The protocol for the real-time PCR was as follows: an initial denaturation step for 3 min at 95°C followed by 40 cycles of 15 s at 95°C, 20 s at 58°C, and 20 s at 72°C. Finally, a melting curve was plotted after each run to verify the product specificity. We used a standard curve for real-time PCR of known copy numbers of total DNA of *Burkholderia glumae* for bacteria and *Amorphotheca resinae* for fungi. This standard curve with serial dilutions was generated to show the proportion between gene copy number and Ct value.

### Sequences Obtained and GenBank Submissions

Raw sequence data have been submitted to the European Nucleotide Archive. They are publicly available under accession PRJEB40662. Assemblies of the six fungi–bacteria metagenomes are available via IMG/MER^2^ using the IMG IDs 3300039030, 3300039916, 3300038809, 3300039918, 3300038808, and 3300039917.

## Results

### Macro- and Microscopic Investigations of Fuel Biofilms

**FIGURE 1:**
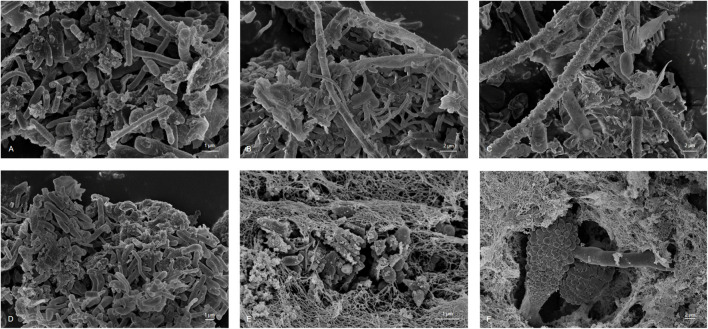
Scanning Electron Microscopy microphotography of sampled material showing hypha and conidia of fungi species and bacteria integrated in an EPS matrix **(A)** sample 1, **(B)** sample 2, **(C)** sample 3, **(D)** sample 4, **(E)** sample 5, and **(F)** sample 6; scale bars of 1–2 μm are indicated in the images (REM LEO 1525, 5.00 kV).

**FIGURE 2:**
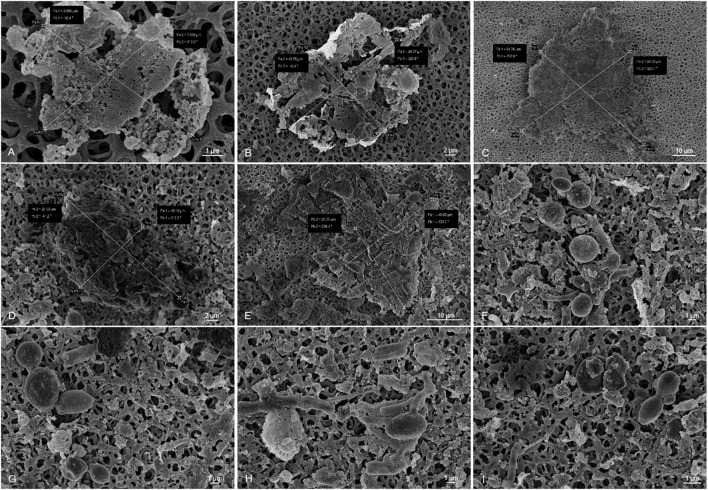
Sonification experiments of filtered kerosene on cellulose acetate membranes (F and W sampling). Sonification conditions: amplitude 60% of 200 W, cycle: 50%, volume: 500 ml, **(A–C)** sonification time: 0 s, **(D–F)** Sonification time: 15 s, **(G–I)** sonification time: 30 s. Scale bars of 1–10 μm are indicated in the images (REM LEO 1525, 5.00 kV).

Biofouling is a result of microbial growth in fuels. Growth occurs at the interface of the fuel and water phase and on the tank surface. Today, only a limited number of detailed studies are available. Therefore, to better understand the 3D structures and makeup of kerosene-grown microbial biofilms, we used microscopical techniques. Scanning electron microscope (SEM) images showed a multilayer and complex structure of the formed biofilms. Further SEM inspections identified various bacterial and fungi-like structures (Figure 1). The morphological differences of the bacteria ranged from spherical to cylindrical forms (rods) with more or less rounded ends. In addition, various extensions and attachment “organs” were visible. In addition, we observed various fungal filamentous structures. Furthermore, we noticed a tight adherence of bacteria to few fungal species. The SEM images also indicated that the cells produced exopolymeric substance EPS and in part net-like or filamentous structures to from lump-like aggregates (Figures 1, 2). Surprisingly, not only in the biofilm sample but also in the liquid samples were we able to detect three-dimensional microbial aggregates (Figure 2). The sizes of these liquid phase multispecies aggregates ranged from 7.69 to 61.26 μm in length and width. Interestingly, the use of sonification allowed the disruption of these aggregates. After a sonification step of up to 30 s, we were able to dissolve particles and separate the microorganisms from their containing EPS matrix.

Altogether, these data implied that the analyzed kerosene-grown biofilms built rather complex 3D structures and that they are found not only on the interphase but also in form of aggregates (organic matter particles, OMPs) in the liquid phase.

### Population Structure, Metabolic Potential, and Most Active Genes of Microorganisms in Fuel-Containing Biofilms

#### Metagenome-Based Microbial Community Analyses of Fuel Biofilms

To further advance the phylogenetic and genome-wide analyses, we established the metagenomes of six fuel samples derived from kerosene tanks of different commercial airplanes. The Illumina-based analyses established DNA contig sequences ranging from 53.9 to 116.2 Mbp, for each of the six metagenomes relying on 19,529 to 53,653 assembled contigs. The largest contigs ranged from 0.3 to 1.5 Mbp, respectively (Supplementary Table 2). Assuming that the genome size of the different bacteria ranges from 3 to 8 Mbp and the main fungi’s genome size is about 28 Mbp, it is reasonable to speculate that the established DNA sequences represent the microbial community’s metagenome significantly.

**FIGURE 3:**
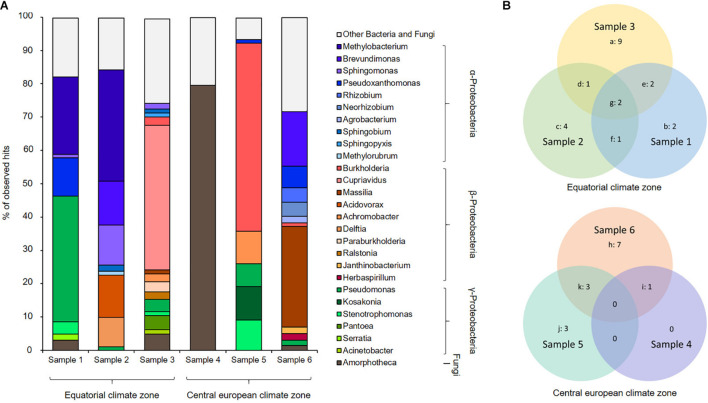
Phylogenetic profiling of fuel biofilm-derived gene sequences based on Kraken2 analysis. **(A)** Metagenome stacked bar chart Kraken2, cutoff 1%, **(B)** Venn diagram showing the number of bins affiliated with assumed organisms uniquely, and the overlap of organisms within particular relationships using the Kraken2 dataset; digits indicate number of organisms a: *Cupriavidus, Acinetobacter, Burkholderia, Paraburkholderia, Sphingopyxis, Pantoea, Massilia, Ralstonia, Achromobacter*, b: *Serratia, Pseudoxanthomonas, c: Acidovorax, Delftia, Methylorubrum, Brevundimonas*, d: *Sphingobium*, e: *Amorphotheca, Stenotrophomonas*, f: *Methylobacterium*, g: *Pseudomonas, Sphingomonas*, h: *Agrobacterium, Herbaspirillum, Rhizobium, Janthinobacterium, Massilia, Neorhizobium, Brevundimonas*, i: *Amorphotheca*, j: *Kosakonia, Stenotrophomonas, Achromobacter*, k: *Pseudomonas, Burkholderia, Pseudoxanthomonas*.

A Kraken2 analysis (Wood and Salzberg, 2014) calculated, under a cutoff of 1% of abundance, a presence of up to 14 microbial genera per metagenome (Figure 3A). Our data imply that the gene sequences were mainly affiliated with members of α-, β-, and γ-Proteobacteria and the Ascomycota. On the genus level, the bacteria *Pseudomonas, Burkholderia, Sphingomonas*, and *Methylobacterium* and the filamentous fungus *Amorphotheca* were the most abundant organisms, while it is important to note that the datasets implied a highly diverse distribution of various genera inside the respective kerosene tanks (Figure 3A).

At least one of the samples, sample 4, appeared to be dominated by fungal sequences affiliated with *Amorphoteca*. For sample 1, we mainly identified *Methylobacterium, Pseudoxanthomonas*, and *Pseudomonas*; sample 2 was mainly composed of *Methylobacterium, Brevundimonas, Sphingomonas, and Acidovorax*. In fact, sample 3 contained primarily *Cupriavidus* but harbored the highest number of different microorganisms, namely, 14 various organisms, whereas the other metagenomes were composed of 1 to 11 different organisms. Sample 5 was dominated by *Burkholderia* but also showed proportions of *Achromobacter, Kosakonia*, and *Stenotrophomonas*, while we identified mainly *Brevundimonas* and *Massilia* for sample 6. Apart from sample 5, all metagenomes contained proportions of the filamentous fungus *Amorphotheca*.

A Venn diagram analysis of the Kraken2 dataset was performed to visualize the commonly harbored and individually distributed genera between the six assembled metagenomes (Figure 3B). Sample 1, sample 2, and sample 3 were collected in the equatorial climate zone and showed a core genome of the genera of *Pseudomonas* and *Sphingomonas*. The genera *Amorphoteca* and *Stenotrophomonas* were shared by sample 1 and sample 3 whereas *Methylobacterium* was detectable in sample 1 and sample 2 and *Sphingobium* in sample 2 and sample 3. The individual distribution of the microbial communities appeared even higher within the three metagenomes collected in the central European climate zone, which are sample 4, sample 5, and sample 6. No core microbiome could be described for the three samples from the central European climate zone. Sample 5 and sample 6 uniquely harbored the genera *Pseudomonas*, *Burkholderia*, and *Pseudoxanthomonas* whereas sample 4 and sample 6 shared DNA sequences from the fungal genus *Amorphoteca*.

Taken together, the Venn diagrams implied that the fuel based-pangenomes harbor a highly diverse gene set based on DNA similarity searches (Figure 3B). However, based on a functional analysis, the functional pangenome was relatively similar. The metabolic properties of the respective habitats did not differ on a larger scale (Tables 1, 2).

**TABLE 1:** Key features of microbial biofilm formation and bio-corrosion in kerosene environments of metagenome samples 1–6 using IMG function search.

	1	2	3	4	5	6
**Transporter, efflux pumps, and secretion systems**						
Transport systems	217	243	205	165	220	224
Efflux pumps	419	547	461	66	353	304
Secretion systems types I to VII	138	224	273	2	183	81
**Kerosene degradation**						
Alkane hydroxylases/monooxygenases	17	35	6	8	22	22
Cytochrome P450	40	26	45	98	50	40
Aldehyde-/alcohol dehydrogenases	288	364	334	214	321	347
**Bacterial biofilm formation**						
EPS and biofilm synthesis	14	11	13	0.9	12	4
Attachment and motility-associated proteins	361	561	517	98	359	463
Quorum sensing system	4	13	4	–	4	3
**Fungal biofilm formation**						
Polymer synthesis	0.6	–	–	4	3	1
Proteins and transcription factors	1	–	2	6	0.9	1
**Bio-corrosion and polymer degradation**						
Formate dehydrogenase	26	46	24	10	33	0.5
Dissimilatory sulfite reductase	–	–	–	–	–	0.5
Lipase	11	6	7	18	12	13
PET-esterase	14	16	14	7	13	13
Potential polyurethanase-like enzymes	76	89	76	37	71	87

*Data shown in total number of hits per 50 Mb.*

**TABLE 2:** Key features observed in the microbial metagenomes of samples 1–6 using COG-based analysis.

	1	2	3	4	5	6
Amino acid transport and metabolism	8.77	8.35	8.23	9.46	9.74	8.48
Carbohydrate transport and metabolism	6.44	5.4	5.28	8.63	6.83	6.52
Cell division and cell wall biogenesis	6.62	6.84	6.79	5.3	6.28	6.86
Cell motility and extracellular structures	2.74	2.76	2.95	1.58	2.6	2.85
Metabolism of cofactors, vitamins, and secondary metabolites	7.49	7.38	7.68	9.85	8.22	7.92
Defense mechanisms	2.51	2.46	2.51	2.94	2.45	2.5
Energy production and conversion	5.94	6.13	6.08	6.48	5.89	5.65
General function prediction only	10.51	9.6	10.45	13.39	11.17	10.78
Transport mechanisms and secretion systems	8.74	9.08	9.41	7.84	8.78	8.34
Lipid transport and metabolism	4.34	4.67	5.14	5.07	4.74	4.83
General DNA/RNA metabolism	5.78	5.79	5.42	5.87	5.04	5.86
Signal transduction mechanisms	5.84	6.22	5.96	4.91	5.3	5.74
Transcription	7.52	7.83	7.33	3.72	7.83	6.51
Translation and posttranslational modification	9.1	8.68	9.24	10.08	8.36	9.74
Unknown and other functions	7.73	8.81	7.52	4.87	6.78	7.42
Not in COG	48.61	32.2	44.28	76.29	47.43	46.51

*Data shown in % of all hits.*

To validate the Kraken2-based phylogeny, an IMG phylogeny was generated based on the phylogenetic distribution of genes using gene count. Furthermore, a 16S rRNA analysis based on the IMG 16S rRNA genes from the metagenomic dataset was generated (Supplementary Table 3). Both additional phylogenies support the Kraken2 analysis. The IMG phylogenetic and IMG 16S rRNA analyses cover the most relevant genera found by Kraken2 up to the respective cutoffs.

#### 16S rDNA Gene and 18S ITS-Spacer Amplicon Analyses of 1,258 Aircraft Swabs

Notably, the relative abundance of the bacteria and fungi cannot be estimated based on the above-described metagenome data sets. For this purpose, quantitative PCR (qPCR) is a better-suited and well-known technology (Sørensen et al., 2011; Leuchtle et al., 2015; Martin-Sanchez et al., 2016; de Azambuja et al., 2017; Radwan et al., 2018). Therefore, we analyzed the abundance of the overall copy number of bacteria and fungi by using specific primers for either the 16S rRNA genes or the 18 S ITS spacer regions. In detail, we used the primers bact_338f/518r and fungi_NL1f/LS2r, which amplify regions of the 16S rRNA and the ITS gene, respectively (O’Donnell, 1993; Ovreås et al., 1997; Cocolin et al., 2000; Ishii and Fukui, 2001). Thereby, it was possible to estimate and validate the bacterial and fungal abundance in various fuel (F), water (W), and biofilm samples (B). In total 1,258 samples (1,008 F-, 110 W-, 140 B-samples) were tested for the general presence of bacteria and fungi. Our analyses implied a thoroughly variable distribution of bacteria and fungi within the different sample types (Supplementary Figure 1). Recurring patterns are observable in bacterial gene copy numbers scaling up to 8,000 in F-samples, up to 80,000 in W-samples, and up to 140,000 in B-samples. Fungi are limited in F- and W-samples, with up to 100 and 22,000 gene copy numbers, respectively, but may be distinctly present in B-samples with up to 100,000 copy numbers in particular samples.

Altogether, these data confirm the relatively high microbial diversity in the different fuel samples. These analyses also confirmed that mainly bacteria affiliated with the genera of *Methylobacterium, Pseudomonas, Sphingomonas, Pseudoxanthomonas, Brevundimonas, Achromobacter, Kosakonia, and Burkholderia* were present and that fungi mostly belonged to Amorphotheca and members of the genera *Aspergillus, Fusarium, and Penicillium*.

#### Metabolic Potential of Microbial Communities

Based on the above made observations, we set out to analyze the metabolic potential of selected kerosene biofilms. Thereby, we analyzed the metagenomes of the above sequenced biofilms.

For the functional analyses of the metagenomes, we used the Pfam, COG, and KEGG databases. The general COG-based analysis highlighted the metabolism of amino acids, carbohydrates, and lipids (Table 2) as well as key features affiliated to transport of ions, cell motility, and extracellular structures, crucial functions for alkane degradation, biofilm formation, and most probably bio-corrosion (Table 1). Searching for genetically coded properties for microbial fuel degradation, biofilm formation, and bio-corrosion, the IMG function search pipeline identified a multitude of genes for alkane hydroxylases, as well as aldehyde- and alcohol dehydrogenases possibly involved in fuel degradation. For possible microbial biofilm formation, we identified a large number of Quorum sensing systems involving proteins, attachment pili and flagella-associated proteins, and genes, which are involved in the synthesis of EPS components. Mechanisms and genes linked to biofouling and corrosion are secreted hydrolases, mono- and di-oxygenases, lipases and various esterases, produced acids (propionate, succinate, acetate, formate, and butyrate), and cation and electron transporters (Table 1).

#### RNA-seq Identifies Active Genes

While metagenome-based studies only describe the genetic potential, no transcriptional studies are available on fuel-affiliated biofilms.

**FIGURE 4:**
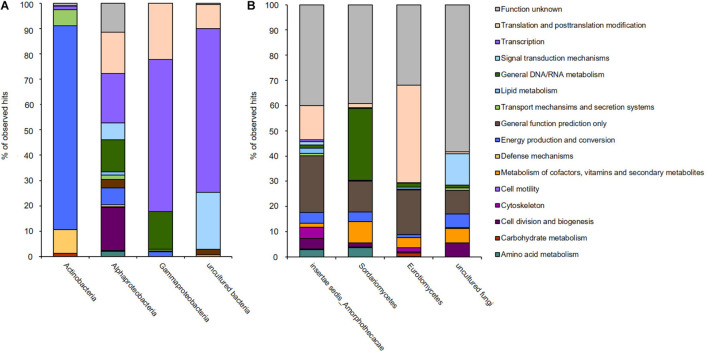
Transcribed microbiome and expressed genes of **(A)** bacterial and **(B)** fungal classes of one biofilm sample from a fuel-containing habitat. The generated sequence data (approximately 132 mio reads) were mapped to the available six metagenomes.

Therefore, and in the light of the above made analyses and observed results, we asked which genes were the most strongly transcribed ones in fuel biofilms. Because of technical reasons and the rather limited access to aircraft fuel tank access, it was, however, not possible to analyze a larger number of fuel biofilm transcriptomes. To obtain a comprehensive overview of all genes that could possibly play an important role in fuel biofilms, we analyzed the transcriptome of a single biofilm sample (Supplementary Table 4 and Figure 4). From this single sample, triplicates were analyzed by RNA-seq. The generated sequence data were mapped to the available six fuel metagenomes. In total, there are 112,137,168 reads mapped to bacteria and 130,184,802 mapped to fungi corresponding to the NCBI datasets. After the filtering of a mapping quality score ≥ 10, there are 36,214,506 reads affiliated with bacterial contigs, and 20,180,691 reads were affiliated with fungal contigs (Supplementary Table 4). The ribosomal proteins of bacteria and fungi are, of course, distinct from the ribosomal rRNAs depleted from (meta)-transcriptomic assays.

**FIGURE 5:**
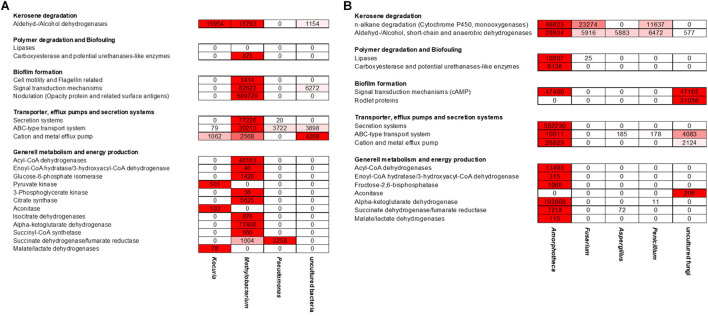
Heat map reflecting the expression of genes affiliated with kerosene and polymer degradation, biofilm formation, transport and secretion systems, and general metabolism and energy production mechanisms of the individual **(A)** bacterial and **(B)** fungi-related reads; color key: 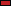 high expression level, 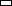 low expression level.

Since the above-analyzed microbial fuel biofilms contained a rather small number of different bacteria, the RNA-seq data covered a significant portion of overall metagenomes. Using these comprehensive data sets, we asked three main questions: (i) first, which are the most strongly expressed genes in fuel biofilms in general? (ii) second, what are the main metabolic routes in these biofilms? and (ii) third, which are the genes and enzymes possibly involved biofouling? (Supplementary Table 5 and Figure 5).

The transcriptionally most active bacterial species were *Methylobacterium* sp. (5,332,547 reads mapped), *Pseudomonas* sp. (176,762 reads mapped), and *Kocuria* sp. (37,444 reads mapped). For fungi, *Amorphoteca* sp. (11,826,916 reads mapped), *Fusarium* sp. (2,521,069 reads mapped), *Penicillium* sp. (962,343 reads mapped), and *Aspergillus* sp. (480,511 reads mapped) were most active on the level of transcription and a high number of reads for other and unknown bacteria and fungi were identified (2,067,530 reads mapped). To identify the most relevant and strongly expressed genes, we decided to focus on the top 250 expressed genes (excluding ribosomal proteins) in our analyses (Supplementary Table 5).

Most notably, however, was that the transcriptome dataset hinted toward a metabolic symbiosis between fungi and bacteria (Figure 5). Thus, in our analyses we identified various genes possibly affiliated with the degradation of C10–C18 carbons of kerosene (e.g., hydrolases and oxygenases) mainly by the fungi up to short-chain carbons, which can then be used in the metabolism of the bacteria. We observed a relatively high number of genes, which related to esterase/lipase activity and which are potentially relevant for long-chain fatty acid degradation. Our datasets determined the metabolic activity of the bacteria up to the citrate cycle. In addition, we identified many bacterial transporters and efflux pumps, which are important for cation/multidrug and metal metabolism. Furthermore, we are able to detect a high number of strongly transcribed genes, which encoded for various secretion systems and cation/multidrug and metal efflux pumps. In addition, we identified genes which are related to cell motility, flagellin synthesis, and adhesion to host structures.

**FIGURE 6:**
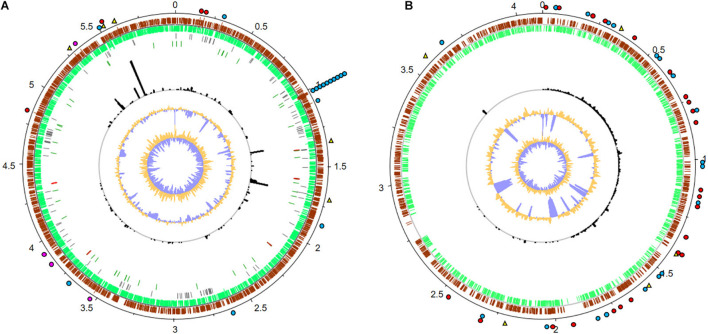
Metatranscriptome mapping to the reference genomes of **(A)** *Methylobacterium brachiatum* (GCF_003697185.1) and **(B)** *Amorphotheca resinae* (GCA_003019875.1). Numbers outside the circles show the genome coordinates in Mbp. Moving inward, the subsequent two rings show CDSs in forward (brown) and reverse (green) strands. Pseudogenes are displayed in gray; genes coding for tRNAs and rRNAs are marked in dark green and red, respectively (*M. brachiatum* only). Black bars indicate RPKM values for each CDS and are displayed in the same scale for **(A,B)**. The last two inner plots indicate GC content and a GC skew [(GC)/(G + C)], where dark yellow and purple indicate values above and below average, respectively. The top 5 most expressed genes are denoted with a yellow triangle. Genes associated with kerosene degradation are marked with a red dot, potential mechanisms involved in polymer degradation with a blue dot, and genes for biofilm formation with a pink dot. For real RPKM values and further details, see Supplementary Table 6.

A more detailed analysis of the *Methylobacterium* and *Amorphotheca*-affiliated transcriptome data identified a number of genes with potential relevance in the degradation of fuel components. For this, transcripts were mapped to the reference genomes of both organisms (Figure 6 and Supplementary Table 6). Expression levels (RPKM) were normalized against rpoD for bacteria and TFIIB genes for fungi, where a log2 fold value above 2.0 was understood as highly expressed (Supplementary Table 6) (Knutson and Hahn, 2013; Gelev et al., 2014; McMillan and Pereg, 2014; Krzyżanowska et al., 2019). In Figure 6, highly expressed genes are marked. Highlighted areas show gene clusters which are highly significant for the analyzed biofilm and habitat.

For *Methylobacterium*, the five most strongly expressed genes are genes which were 5-formyltetrahydrofolate cyclo-ligase, efflux transporter permeases, and hypothetical proteins. Further, we identified many transcriptionally active gene clusters (3,574 total mapped reads) coding for a multitude of oxidoreductases, dehydrogenases, and proteases (Figure 6). In addition, a remarkable number of genes are involved in hypothetical proteins. Notably, a large number of reads (5,947) mapped to genes code for aldehyde dehydrogenases and alkene reductases. Furthermore, a total number of 875 reads are involved in alginate biosynthesis and flagella-related proteins, which are relevant for biofilm formation (Supplementary Table 6).

Despite the poor level of annotation of the reference genome of *Amorphotheca*, a protein homolog with a proper annotation could be found for more than 80% of all the CDS. In the available dataset, the five most strongly transcribed expressed genes were marked as hypothetical proteins. In addition, a total of 15,441 RNA reads are mapped to potential aldehyde dehydrogenases and cytochrome P450-like enzymes. These are potentially involved in oxidative processes that could be linked to kerosene degradation. In addition, more than 4,322 reads are mapped to proteins annotated as carboxylesterase type II and putative lipases or esterases that could potentially be involved in hydrolysis of various ester bonds (Figure 6). In addition, and because of the relatively high number of nodulation and surface antigen-regulated genes (3,450 total reads mapped, Supplementary Table 6) which are linked to biosynthesis of biofilms and general metabolism, one can speculate that the biofilm lifestyle is of strong importance for growth and survival in kerosene- and fuel-containing habitats. This is a significant point for additional effects of polymer degradation and biofouling processes. These enzymes are significant for the analyzed habitat and for implementing surviving strategies of the organisms and a possible symbiotic interaction between fungi and bacteria.

In summary, these data give forth a hint on the overall metabolism of a fuel biofilm on a metagenome and transcriptome level.

## Discussion

A comprehensive understanding of the composition of the microbial community, the grade of microbial contamination, and its functional properties is a requirement for controlling and reducing microbial proliferation in fuel-containing habitats.

In this study, we mainly used next-generation sequencing and qPCR strategies to analyze the fungi–bacteria interaction and physiological properties in more detail. With respect to our phylogenetic analysis and in addition to previous studies, our data imply that the main microbial contaminants were mainly affiliated with members of α-, β-, and γ-Proteobacteria and Ascomycota. In general, several microorganisms, including bacteria, filamentous fungi, and yeasts are able to degrade n-alkanes. Among these microorganisms, medium-length-chain n-alkane degraders have dehydrogenases, cytochrome P450, and monooxygenases (Whyte et al., 2002; Rojo, 2009; Das and Chandran, 2011; Jurelevicius et al., 2013). Many of the generated contigs of the metagenome datasets code for known mechanisms involved in the degradation of kerosene.

Our metagenome and transcriptome datasets support in part this hypothesis and indicated that in general very common processes are expressed. The most active bacteria observed within the fuel biofilms were *Methylobacterium, Kocuria*, and *Pseudomonas* as well as uncultured and unknown bacteria. *Methylobacterium* sp. are a strictly aerobic, facultatively methylotrophic, fastidious, slow-growing bacteria isolated from various environments (i.e., leaf surfaces, soil, dust, and fresh water) (Patt et al., 1976; Rice et al., 2000; Sanders et al., 2000; Kovaleva et al., 2014). *Methylobacterium* is a strong biofilm producer and exhibits tolerance to cleaning and disinfecting agents and to high temperatures (Simões et al., 2010; Yano et al., 2013; Beale et al., 2014). Due to its slow growth, the bacterium can be easily missed during culture-dependent screening. *Kocuria* is a genus of Gram-positive bacteria in the phylum Actinobacteria and belongs to the family Micrococcaceae. It grows in a wide range of ecological niches (Stackebrandt et al., 1995; Park et al., 2010). SEM images of biofilms formed by *K. rhizophila* are composed of amassed cocci and fibriform extracellular matrix of proteins and carbohydrate (Kavitha and Raghavan, 2018). *Pseudomonas* sp. is a ubiquitous Gram-negative bacterium, capable of adapting to versatile environments such as human tissues and environmental surfaces (Mesaros et al., 2007; Winsor et al., 2011; Percival et al., 2015). Members of the genus *Pseudomonas* are well-known biofilm-forming bacteria and naturally resistant to a wide range of antibiotics, making antibiotic and disinfection treatments ineffective (Drenkard and Ausubel, 2002; Sillankorva et al., 2008; Chang, 2018; Maurice et al., 2018).

With respect to the assumed bio-corrosion potential, we observed a rather low number of dissimilatory sulfite reductase (DsrA, DsrB) genes, which participate in the last step of dissimilatory sulfate reduction to generate sulfides, a mechanism that was shown to induce corrosion of iron and steel (King and Miller, 1971). Based on the above results, we hypothesize that microbia-influenced corrosion (MIC) by sulfate-reducing bacteria (SRB) are potentially not of high relevance in the analyzed samples.

Instead, we were able to detect a significant number of bacterial and fungal genes and transcripts, which are related to biofilm formation, cation and metal efflux pump transport, and acid production. Notably, heterogeneous microbial structures, primarily formed by aerobic and facultative anaerobic microorganisms, lead to creation of oxygen gradients due to oxygen/nutrient consumption and pH gradient formation (Picioreanu and van Loosdrecht, 2002). The different concentrations of dissolved oxygen lead to cathodic and anodic areas where, e.g., metal is oxidized to ions in anodic areas and dissolved oxygen is reduced in cathodic areas (Vargas et al., 2017). Thereby, EPS binds free metal ions, which further stimulates the anodic dissolution of metal (Beech and Sunner, 2004). Overall, an increasing production of EPS in biofilms leads to destruction of protective oxide films in technical systems, thereby promoting corrosion (Chen and Zhang, 2018). Within this framework, it is notable that formate dehydrogenase further contributes to corrosion. The transmembrane domain of the beta subunit consists of a single-transmembrane helix. This domain acts as a transmembrane anchor, allowing the conduction of electrons within the protein. In combination with biofilm formation, an induction of corrosion via direct metal–microbe electron transfer could be a major point for MIC (Jormakka et al., 2002; Beech and Sunner, 2004; Deutzmann et al., 2015; Tang et al., 2019).

While current corrosion research has mainly focused on the tank or fouling of the fuel, scarce information is yet available on the corrosion of the coating used to protect the tank material. These protective paintings are many polyurethane (PUR)-based polymers (Seymour and Kauffman, 1992). Our data imply that many enzymes especially lipases and other hydrolases may act on these protective paintings and contribute to the corrosion. Thereby, it is well known that lipase/esterase-like (including carboxylesterase) and polyurethanase-like enzymes are able to attack and decompose various polyester compounds including coatings and sealant material (Russell et al., 2011; Danso et al., 2018, 2019; Kawai et al., 2019).

The most prominent fungal representative in these selected fuel samples was *Amorphotheca*. Members of the genera *Aspergillus, Fusarium*, and *Penicillium* as well as other and unknown fungi were also confirmed to be active fungal contaminants (Darby et al., 2001). *Amorphotheca*, widely known by the anamorph name *Hormoconis resinae* (Lindau) Arx & G.A. de Vries or its obligate synonym *Cladosporium resinae* (Lindau) G.A. de Vries, grows in hydrocarbon-rich substrates such as jet fuel, cosmetics, and wood preserved with creosote or coal tar (Parbery, 1969; Seifert et al., 2007).

Many metabolites produced by fungi, which include formic, citric, and acetic acids, are damaging to metals, glass, masonry, and other materials and therefore contribute to corrosion (Little et al., 2001; Gu, 2003; de Leo et al., 2013; Flemming, 2020). The organic acids produced by fungal species may contribute significantly to microbiologically influenced corrosion. Bio-corrosion of aluminum and its alloys has been attributed to contamination of jet fuels caused by the fungi *Amorphotheca resinae, Aspergillus fumigatus, Penicillium corylophilum*, and *Fusarium oxysporum* (Teh and Lee, 1973). *Amorphotheca resinae* utilizes the hydrocarbons of fuel to produce organic acids; the large quantities of organic compounds excreted by this fungus are capable of causing bio-corrosion of storage tanks and transporting pipelines (Flemming and Geesey, 1995). Other fungi detected that may contribute to bio-corrosion of oil pipelines, blockage of pipes, valves, and filters, and incorrect reading of fuel probes include *A. fumigatus* which is involved in iron reduction from ferric state (Fe^3+^) to ferrous (Fe^2+^) state. *Aspergillus terreus* produces aspartic acid, a product of general metabolism, which at high concentrations will contribute to bio-corrosion of oil pipelines (Haas, 2003). Each fungus presents a different pattern of biofilm development, spore adhesion, and monolayer and EPS production (de Siqueira, 2015), which may be involved in several functions, including adhesion to host proteins and cells, dispersion by air currents, and protection against chemicals, enzymes, and phagocytic cells (Beauvais et al., 2007; Mowat et al., 2009; Siqueira and Lima, 2012).

Within this framework, our findings imply that at least some of the triggers and signals involved in the microbial interaction with fungi and bacteria are already of relevance in industrially and biotechnologically used systems. In summary, the current study gives a detailed insight into the metagenomes and partial transcriptome of fuel microbial communities. Future work will now have to unravel the detailed interaction between the bacteria and eukaryotes in different aspects of biofouling.

## Data Availability Statement

Raw sequence data have been submitted to the European Nucleotide Archive. They are publicly available under accession PRJEB40662. Assemblies of the six fungi bacteria metagenomes are available via IMG/MER (https://img.jgi.doe.gov) using the IMG IDs: 3300039030, 3300039916, 3300038809, 3300039918, 3300038808, and 3300039917.

## Author Contributions

IK, LB, and YH contributed to experimental design; lab work of phylogenetic, metagenomic, and transcriptomic approaches; and writing of the research article. MQ, MA, and PP-G contributed to assembly of metagenomic and transcriptomic datasets and bioinformatic approaches. DI contributed to lab work on metagenomic and phylogenetic approaches. EK contributed to lab work of transcriptomic approaches. BH, TL, CS and RR contributed to the delivery of sample material for analysis and lab work for sonification processes. WS contributed to general experimental design and writing of the research article. All authors contributed to manuscript revision, read, and approved the submitted version.

## Conflict of Interest

BH, TL, CS, and RR were employed by company Lufthansa Technik AG HAM. The remaining authors declare that the research was conducted in the absence of any commercial or financial relationships that could be construed as a potential conflict of interest.

## Publisher’s Note

All claims expressed in this article are solely those of the authors and do not necessarily represent those of their affiliated organizations, or those of the publisher, the editors and the reviewers. Any product that may be evaluated in this article, or claim that may be made by its manufacturer, is not guaranteed or endorsed by the publisher.

## Footnotes

1
https://www.ncbi.nlm.nih.gov/refseq/about/nonredundantproteins/

2
https://img.jgi.doe.gov

## Supplementary Material

Supplementary Figure 1 Gene copy-number quantification, primer 338/518 for bacteria and NL1/LS2 for fungi, minimum cutoff 1000 copies. color key:  copy number/ng DNA for bacteria,  copy number/ng DNA for fungi, **(A)** for selected fuel samples (F_01-F_14), **(B)** for different water samples (W_01-W_22), **(C)** for various biofilm samples (B_01-B_28).

Supplementary Table 1 Sample collection of the six samples for the metagenome analysis, the transcriptome analysis and the used 1258 samples for the qPCR analysis. Samples were collected and processed as described in section “Materials and Methods.” Samples were collected and transported to the laboratory and kept at 4° until processing.

Supplementary Table 2 Overall numbers of sequences and contigs generated for microbial communities of sample 1–6 and IMG metagenome statistic.

Supplementary Table 3 Phylogenetic profiling_Comparison between Kranken2, IMG Phylogeny and 16S rRNA Hits analysis.

Supplementary Table 4 Overall numbers of sequences and contigs generated for the transcriptome.

Supplementary Table 5 Top 250 expressed genes in the biofilm sample.

Supplementary Table 6 Transcriptome map representing the genome-wide RNA-seq data for *Methylobacterium* (GCF_003697185.1) and *Amorphotheca* (GCA_003019875.1).
