# Supplementary figures and images for: Deep (Meta)genomics and (Meta)transcriptome Analyses of Fungal and Bacteria Consortia From Aircraft Tanks and Kerosene Identify Key Genes in Fuel and Tank Corrosion
Source: Front Microbiol. 2021 Oct 1;12:722259. doi: 10.3389/fmicb.2021.722259 (PMC8525681; doi:10.3389/fmicb.2021.722259)

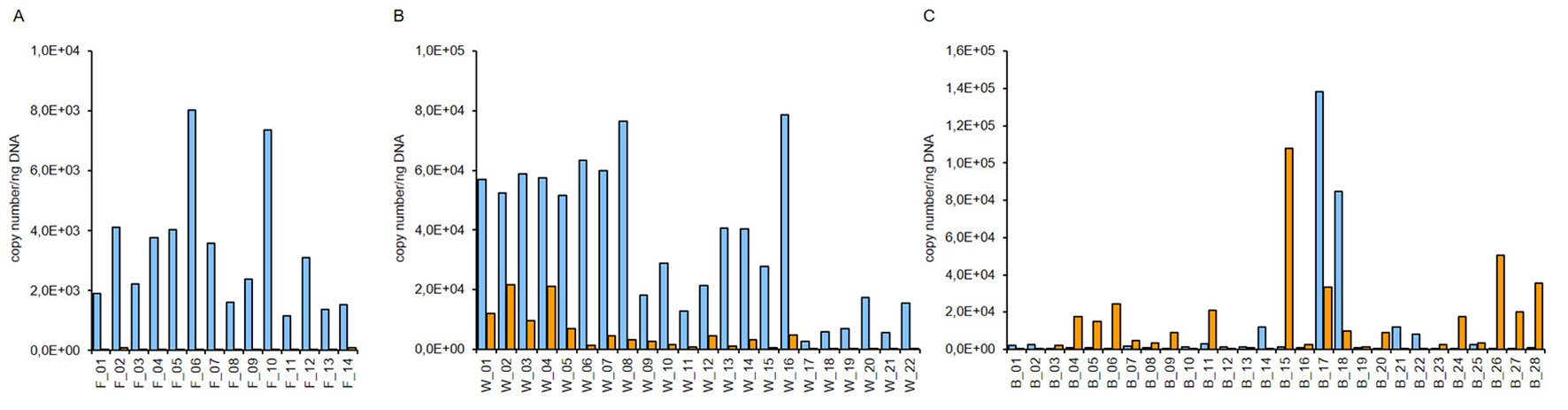

Supplement: Supplementary Figure 1 — Gene copy-number quantification, primer 338/518 for bacteria and NL1/LS2 for fungi, minimum cutoff 1000 copies. color key: copy number/ng DNA for bacteria, copy number/ng DNA for fungi, (A) for selected fuel samples (F_01-F_14), (B) for different water samples (W_01-W_22), (C) for various biofilm samples (B_01-B_28). [file Image_1.jpeg]
