# Supplementary material for: Deep (Meta)genomics and (Meta)transcriptome Analyses of Fungal and Bacteria Consortia From Aircraft Tanks and Kerosene Identify Key Genes in Fuel and Tank Corrosion
Source: Front Microbiol. 2021 Oct 1;12:722259. doi: 10.3389/fmicb.2021.722259 (PMC8525681; doi:10.3389/fmicb.2021.722259)
Supplement: Supplementary Table 2 — Overall numbers of sequences and contigs generated for microbial communities of sample 1–6 and IMG metagenome statistic. [file Table_2.docx]

**Supplemental TABLES**

**Supplemental TABLE 2**: Overall numbers of sequences and contigs generated for microbial communities of sample 1-6 and IMG metagenome statistic

|  | **1** | **2** | **3** | **4** | **5** | **6** |
| --- | --- | --- | --- | --- | --- | --- |
| **Reads Illumina (filtered)** | | | | | | |
| Total no. | 43,563,718 | 49,118,632 | 42,487,831 | 32,856,993 | 140,479,091 | 167,777,531 |
| Average length (bp) | 150.274 | 148.468 | 144.794 | 144.100 | 149.096 | 223.173 |
| Duplicates (%) | 59 | 38 | 54,5 | 29 | 58 | 74 |
| Fails (%) | 9 | 0 | 27 | 18 | 9 | 25 |
| GC (%) | 60 | 65 | 59 | 46 | 65 | 61 |
| **Contigs-assembly (Spades)** | | | | | | |
| No. | 27,016 | 19,529 | 53,653 | 24,075 | 47,117 | 20,623 |
| Total length (bp) | 84,557,395 | 61,418,381 | 114,420,529 | 53,927,617 | 116,178,713 | 94,370,133 |
| No. ≥ 1000 bp | 14,245 | 9,934 | 23,330 | 7,600 | 18,343 | 7,773 |
| N50 size (bp) | 9,430 | 15,729 | 4,559 | 24,130 | 19,657 | 30,522 |
| Largest (bp) | 281,266 | 495,115 | 444,773 | 275,105 | 835,736 | 1,496,958 |
| GC (%) | 57.31 | 65.52 | 58.78 | 47.71 | 59.99 | 60.38 |
| **IMG ID** **metagenome statistic** | | | | | | |
| IMG ID | 3300038809 | 3300038808 | 3300039918 | 3300039030 | 3300039917 | 3300039916 |
| Number of bases | 75,743,064 | 54,692,720 | 93,871,441 | 42,450,068 | 96,316,795 | 85,980,754 |
| GC count [%] | 57.70 | 65.87 | 58.68 | 47.90 | 61.22 | 60.76 |
| Number of protein coding genes | 85,616 | 61,472 | 114,275 | 48,243 | 104,895 | 92,886 |
| % of assembled protein coding genes | 98.61 | 98.80 | 98.84 | 98.67 | 98.94 | 98.74 |
