# Supplementary material for: Deep (Meta)genomics and (Meta)transcriptome Analyses of Fungal and Bacteria Consortia From Aircraft Tanks and Kerosene Identify Key Genes in Fuel and Tank Corrosion
Source: Front Microbiol. 2021 Oct 1;12:722259. doi: 10.3389/fmicb.2021.722259 (PMC8525681; doi:10.3389/fmicb.2021.722259)
Supplement: Supplementary Table 4 — Overall numbers of sequences and contigs generated for the transcriptome. [file Table_4.docx]

**Supplemental TABLES**

**Supplemental TABLE 4:** Overall numbers of sequences and contigs generated for the transcriptome.

| **Transcriptome sample_value** | |
| --- | --- |
| Reads Illumina (filtered) | |
| Total no. | 242,321,970 |
| Average length (bp) | 65 |
| Duplicates (%) | 85 |
| Fails (%) | 27 |
| GC (%) | 54 |
| Contigs-assembly (Spades) | |
| No. | 7,212 |
| Total length (bp) | 6,082,092 |
| No. ≥ 1000 bp | 1,565 |
| N50 size (bp) | 845 |
| Largest (bp) | 6,881 |
| GC (%) | 51.74 |
